# ‘You can’t just hit a button’: an ethnographic study of strategies to repurpose data from advanced clinical information systems for clinical process improvement

**DOI:** 10.1186/1741-7015-11-103

**Published:** 2013-04-10

**Authors:** Cecily Morrison, Matthew Jones, Rachel Jones, Alain Vuylsteke

**Affiliations:** 1 Engineering Design Centre, University of Cambridge, Cambridge, CB2 1PZ, UK; 2 Judge Business School, University of Cambridge, Cambridge, CB2 1AG, UK; 3 Instrata Limited, 12 Warkworth Street, Cambridge, CB1 1EG, UK; 4 Anaesthesia and Intensive Care, Papworth Hospital NHS Foundation Trust, Cambridge, CB23 3RE, UK

**Keywords:** Clinical information system, Clinical process improvement, Computerized medical records systems, Intensive care, Qualitative research, Secondary use of data, User-customization

## Abstract

**Background:**

Current policies encourage healthcare institutions to acquire clinical information systems (CIS) so that captured data can be used for secondary purposes, including clinical process improvement. Such policies do not account for the extra work required to repurpose data for uses other than direct clinical care, making their implementation problematic. This paper aims to analyze the strategies employed by clinical units to use data effectively for both direct clinical care and clinical process improvement.

**Methods:**

Ethnographic methods were employed. A total of 54 contextual interviews with health professionals spanning various disciplines and 18 hours of observation were carried out in 5 intensive care units in England using an advanced CIS. Case studies of how the extra work was achieved in each unit were derived from the data and then compared.

**Results:**

We found that extra work is required to repurpose CIS data for clinical process improvement. Health professionals must enter data not required for clinical care and manipulation of this data into a machine-readable form is often necessary. Ambiguity over who should be responsible for this extra work hindered CIS data usage for clinical process improvement. We describe 11 strategies employed by units to accommodate this extra work, distributing it across roles. Seven of these motivated data entry by health professionals and four addressed the machine readability of data. Many of the strategies relied heavily on the skill and leadership of local clinical customizers.

**Conclusions:**

To realize the expected clinical process improvements by the use of CIS data, clinical leaders and policy makers need to recognize and support the redistribution of the extra work that is involved in data repurposing. Adequate time, funding, and appropriate motivation are needed to enable units to acquire and deliver the necessary skills in CIS customization.

## Background

The data amassed in the databases of electronic patient records and other clinically-oriented information systems (CIS) can be a valuable resource for purposes other than direct clinical care. Many governments have been promoting secondary usage of this data [1,2]. The key thrusts of these policies have been to encourage the acquisition of CIS by healthcare institutions, and to seek health benefits from the derived data [3,4]. One focus of these policies is for healthcare institutions to use the data that they acquire during clinical care to support clinical process improvement through clinical and service audits (hereafter referred to as audit).

A recent major systematic review, however, has highlighted the unacknowledged work needed to transform the data into an appropriate form for secondary use [5]. Studies that describe case examples of this extra work illustrate that what constitutes ‘good’ data in one circumstance, such as clinical care, differs from what constitutes it in another, such as audit [6,7]. Extra work is therefore required to repurpose the data to be usable in its new context [8-10]. Tension can develop over who should be responsible for this extra work, which can make it difficult to realize the expected benefits on which the policy initiatives are based [5-7].

Advanced CIS allow specially trained local health professionals to customize them and to extract data from their databases [11]. Such capabilities allow units to experiment with different ways of collecting and using data for both direct clinical care and clinical process improvement, iteratively developing strategies to reduce tension between the two. Data-intensive clinical units, such as those that treat critically ill patients, have been at the forefront of using advanced CIS and are particularly suitable to gain insight into this topic.

This study used ethnographic methods to explore how five intensive care units (ICUs) with advanced CIS minimize this tension in order to use data effectively for both direct clinical care and for clinical process improvement. We aimed to characterize the difficulties units face in fully using their CIS data and present the strategies they employ to address these issues. We reflect upon the implications of our findings for both health professionals and policymakers, discussing how they may be generalized to other health settings.

## Methods

### Setting

Five English ICUs that had implemented user-customizable CIS, four in the East of England and one in London, were recruited to the study. Units were chosen to maximize the diversity of CIS vendors and years of ownership, as this was expected to increase the number of strategies observed. As shown in Table 1, units used CIS from four different vendors and had owned them for between 3 and 10 years.

**Table 1:** Description of recruited units

**Clinical information system (CIS) brand**	**Years owned**	**No. of beds**	**Customization time allocated**
Innovian (Draeger)	3	14	3 h/week consultant
Metavision with purchased database (iMDsoft)	4	20	6 h/week nurse plus consultant time
Metavision with own database (iMDsoft)	4	26	Part-time administrator
QS (General Electric)	8	13	Full-time nurse administrator
Carevue (Philips)	10	88	2 full-time nurse administrators

### Data collection

Explicit and implicit data handling and manipulation related to direct clinical care as well as audit were captured using contextual interviews [12]. Contextual interviews, which mix interviews with observation, involve the researcher observing work while it is being carried out in its normal setting, and initiating discussion of particular choices as they are made. Contextual interviews are considered better than interviews in revealing implicit work as they do not rely solely on self-reporting [13].

Given the safety-critical nature of ICU work, the contextual interview method was modified in order to minimize the disruption to health professionals. The contextual interviews were held either in a spare bedspace in the ICU, or, if this was not available, in a nearby office with a CIS. Interviewees were asked to select a recent patient that they had been caring for and to demonstrate how they had carried out specific activities, such as reviewing a patient, earlier that day. The researcher observed how the activity was carried out and probed the interviewee about the choices they made. For example, the researcher might ask a junior doctor, ‘What prompted you to leave those parts of the form blank?’

Contextual interviews were carried out with individuals in each role of the multidisciplinary team as well as any CIS-specific roles to ensure that similar situations were seen from different perspectives [14]. Topic guides were used. These included appropriate tasks in the areas of patient review, documentation, prescribing and secondary uses of data by health professionals. These were chosen to cover the most common day-to-day tasks, derived from observation of care and consultation with health professionals. The topic guides for those with CIS or leadership roles focused on CIS and unit management.

The contextual interviews were carried out by one of the authors (CM), a trained social scientist, and lasted 30 minutes to 1 hour. Interviews were audio recorded and computer screenshots taken to document system use. Additional time was spent observing clinical practices on each unit to contextualize the interview data and support interpretation. Detailed field notes were kept for each observation. These contained descriptions of general ward activities as well as specific episodes of interaction, including discussions between health professionals, informal conversation between the researcher and health professionals, as well as sequences of events during care activities. Data collection took place over a period of 6 months.

### Data analysis

The method of data analysis followed the principles articulated in [15,16] for interpretive evaluations of clinical information systems using ethnographic methods. Interviews were transcribed and loaded into Nvivo 9, [17] a qualitative analysis software package, for review and analysis. Two authors (CM and RJ) first identified all discussions relating to difficulties for those manipulating or collecting CIS data. This provided an initial picture of the types of issues units faced. A second round of analysis aimed to provide greater context to these issues by focusing more broadly on data that encapsulated actions that contributed to or addressed the difficulties.

The identified data was integrated with the field notes from the hours of observation and woven together into a case study for each unit, as common in multisite qualitative research [8,18]. Each case study contained a description of the unit’s CIS and its capabilities, examples of difficulties as seen from multiple perspectives, and strategies used to address these difficulties. This case study approach allowed us to take a unit-wide perspective, establishing the interrelations of roles and how choices of a person in one role may affect someone in another role.

The case studies were compared across units to ascertain common themes. This was performed through extracting the difficulties faced and the resulting strategies onto post-it notes and grouping them according to commonalities. Special attention was paid to finding examples that might contradict or broaden the interpretation of the themes apparent in the formed groups. As findings emerged, groups were iteratively rearranged following the identification of relevant examples from the case studies, discussion among the authors, and review of the original data.

### Ethics and governance

The study was deemed as constituting system development by the Cambridgeshire 1 NHS Research Ethics board and therefore did not require ethical approval. All interviewees were given an information sheet and were asked for verbal consent.

## Results

A total of 54 contextual interviews were carried out across the 5 units, supported by a further 18 h of observation, as described in Table 2. It emerged from the analysis that the clinical leads in all units identified the inability to use their CIS databases for secondary analysis as a key problem. Yet, at the time of the interviews, all units were able to use at least some data for audit. We have organized the results section around the two questions this raised: (1) what are the difficulties that units faced in utilizing their CIS data for audit? And (2) what strategies do they employ to overcome these difficulties?

**Table 2:** Summary of interviews carried out

**Unit**	**No.# of interviews**	**Roles**	**Hours of observation**
1	9	Nurse: HCA, B5, B7, Charge	2
		Doctor: Con/Lcust, SHO	
		AHP: Diet, Pharm, Physio	
2	11	Nurse: HCA, B5, Research	4
		Doctor: Con, SHO, SpR	
		AHP: Diet, Pharm, Physio	
		Other: Clin-lead, Lcust	
3	10	Nurse: B5, B6,	1
		Doctor: Con	
		AHP: Diet, Pharm, Physio	
		Other: Clin-lead, Lcust, IT manager, Audit nurse	
4	12	Nurse: B5, B6, Charge	3
		Doctor: Con, SHO, SpR	
		AHP: Diet, Pharm, Physio	
		Other: Clin-lead, Lcust (nurse) and (doctor)	
5	12	Nurse: B5, B6, Charge	8
		Doctor: Con, SHO, SpR	
		AHP: Diet, Pharm, Physio	
		Other: Clin-lead, Lcust,	
Total	54		18

AHP, allied health professional; HCA, healthcare assistant; B5/6/7, band 5/6/7 nurse; charge, charge nurse; Clin-lead, clinical lead; Con, consultant; Lcust, local customizer; SpR, specialist registrar; SHO, senior house officer; Diet, dietician; Pharm, pharmacist; Physio, physiotherapist.

### What are the difficulties?

We identified 23 situations in which difficulties arose when repurposing CIS data for audit. These ranged from data not being entered by a nurse for a particularly ‘busy’ patient to clinical customizers having to undertake several iterations of data manipulation in another software program to convert the data into an appropriate form for audit. All of the difficulties identified in our data derived from two sources: (1) a need to enter data that would not normally be required for direct clinical care, or (2) a need to reformat data into a machine-readable form. In the first case, health professionals might need to augment or quality control clinical care data, or enter additional data. In the second case, data may not be specifically recorded, appropriately formatted, or have consistent terminology, making it difficult for the software to interpret it even if readily accessible to health professionals. Examples are provided in Table 3, with supporting data offered in Additional file 1.

**Table 3:** Examples of work involved in repurposing data categorized by the problem it illustrates and its source

**Example**	**Problem**	**Source**
Doctors are asked to enter diagnosis at the time of a patient’s admission. Diagnosis is not always known at this time and of little importance to the doctors, who base their work on active issues. For this reason, it often does not get filled in correctly or at all. Its completion is an augmentation of data needed for direct clinical care.	Data entry needs augmenting	Data entry not needed for direct clinical care
Junior doctors are asked to enter past medical history twice: into the admission form and into an audit form. They find this frustrating and time consuming and assign it a low priority with the ramification that it does not always get done.	Duplicate data entry needed	
Nurses are asked to validate measures captured automatically from the monitors. This ensures that values are accurate and not distorted (for example, because the patient moved). Their accuracy is of little value to nurses who can clearly see they are wrong and the correct values adjacent. They do not always correct mistakes, especially if busy. This causes considerable problems at audit time when highest and lowest parameters are searched for.	Quality control of data needed	
An audit for septic patients is not possible because it is not a diagnosis. The computer can only identify those with bacterial pneumonia as a main diagnosis. In contrast, a nurse can easily tell if a patient is septic from looking at the clinical record.	Data not specifically recorded	Data not machine-readable
Clinicians prefer to write their notes in free text to more aptly express the issues of focus to their colleagues. This can make diagnosis, problems, and actions difficult to extract from CIS data.	Data not in appropriate format	
Doctors use a range of terminology for common problems. For example, out of hospital arrest can be termed: cardiac arrest, cardiac standstill, cardiac asystole, and ventricular fibrillation. This makes it very difficult for the computer to search for data related to out of hospital arrest as it cannot assimilate all of the related terms easily as a doctor could do.	Terminology not consistent	

Supporting data is provided in Additional file 1, CIS, clinical information system.

Each of these situations requires extra work for someone. Yet, there can be ambiguity about who is responsible for this extra work as illustrated in the quotations below.

We started to talk about secondary uses of data, which he considered the next big step in the unit. At one point in the conversation, he shook his head forlornly and complained that the ‘juniors’ just didn’t enter the data, which made the whole thing more difficult. -- Field notes (clinical lead)

‘Admitting a patient on this system is a very tedious process…it requires you to input lots of data which to us clinically is not very relevant.…This bit in red, enter past medical history, ICNARC data, so ICNARC is this auditing thing, so I've never seen it and it takes ages to input it on that particular thing but you can't see it on the clinical thing.’-- Junior doctor

‘I don't always take their word for it. I always check it because… people put the wrong information in.’ -- Clinical customizer

In this example, the clinical lead is keen to repurpose the CIS data for audit purposes, but does not take into consideration the extra work this imposes on the junior doctor. The junior doctor assigns a low priority to the non-clinical activity of entering extra data for audit, leaving the clinical customizer to check the data in order to ensure its quality for audit. In each case the responsibility for the extra work is passed on, with much tension over each person’s duty. The strategies employed by the units addressed this extra work and the ensuing tension.

### What strategies were employed?

We identified 11 strategies which units employed to reduce the tension created by the extra work in order to effectively repurpose clinical data for clinical process improvement. Seven of those strategies focused on motivating data entry through reducing it or making it more relevant to care. The other four strategies focused on the issue of data not being in a machine-readable format. All strategies attempted to spread the burden of the extra work. Below we describe each of these strategies and highlight the different roles involved in carrying them out.

#### Automating data entry

All units used automated transfer of data from the vital sign monitors and sometimes other machines, such as ventilators, to the CIS. Although this feature was appreciated and supported good data capture, it required oversight. The interface between monitors and the CIS needed to be developed, updated, and fixed when not working. Someone also had to carry out quality control on the data to remove inaccurate values, such as abnormal readings due to patient movement during measurement. This was delegated to the nurse during the validation process, but the interviews suggested it was more frequently performed by the person producing an audit.

#### Providing smart forms

Smart forms use technical means to reduce data entry. In one unit, a smart form limited the questions asked based on previous answers, and automatically calculated scores. The introduction of this smart form increased data entry 100-fold over the course of a month. Other smart forms shared data between fields, reducing the need for double data entry by junior staff, who, for example, may need to enter past medical history into the CIS for clinical purposes and then re-enter much of the same data into an audit form. Although smart forms are useful for reducing the fatigue and frustration caused by double data entry, many CIS in the study did not have these capabilities or the customizers did not have the skill to program them.

#### Integrating data entry into the workflow

Units put significant effort into increasing data entry through integrating data collection into the workflow. The simplest way this was achieved was by the strategic placement of data fields. One unit increased data entry for a delirium clinical audit by placing it on the neurological care documentation page, making it a part of care rather than a separate audit. Prompts triggered after an event, such as deviation from the platelet infusion protocol, provide another mechanism to enter data at the relevant time. Whole care process timing was also considered. Diagnosis, frequently missing from the CIS record but crucial to many audits, was collected in one unit at discharge when diagnosis is most likely known, increasing entry. The skillful integration of data entry into the workflow requires substantial insight into the workflow by the customizers, as well as thoughtful planning and customization skills.

#### Enabling personal benefit from data entry

Motivating individuals to enter data, through emphasizing personal gain, was still necessary despite the above measures. One unit, for example, adopted a positive approach, naming the people who most consistently entered sepsis data. After telling everyone that those not named needed to improve, compliance increased dramatically. It was argued that this was more effective than naming and shaming tactics. Other units tried to make data personally relevant as a way to encourage entry. For example, several units supplied junior doctors leaving the unit with a listing (for their medical curriculum vitae) of the number and kind of procedures that the CIS had recorded them as having carried out. These approaches require some effort on the part of the customizers.

#### Prioritizing data entry

Inevitably, frontline care priorities outweigh data entry at certain times. Units helped health professionals in making judgments about the need for data entry by prioritizing some data fields. Some units did this by making particular fields, such as weight, mandatory; others added tasks, such as teeth brushing, to the prescription chart. The units that took this approach noted that it had to be used sparingly to ensure 100% completion of accurate, as opposed to false, data. Units also established priorities in data collection by not allowing the documentation to balloon. One unit had a policy of removing a data element for every new one added. These choices were most commonly made by the customizers and required in-depth knowledge of the way the unit functioned on a day-to-day basis.

#### Increasing awareness of data usage

Data entry was also motivated through increasing awareness of the benefits of data usage. For example, one unit took part in a program to help minimize central venous catheter associated bloodstream infections. As part of this, staff were educated about proper care procedures and then data fields were added to the CIS so that the care goals could be assessed. Data collection in this case became part of a larger multidisciplinary discussion about care delivery that encouraged health professionals to attend to certain aspects of care. Aggregate data from this project was then sent to staff on a monthly basis to demonstrate that the unit was improving its practice. The creation of standardized programs can support such initiatives, but still leaves much work for those who implemented them in the unit, often clinical customizers.

#### Emphasizing data entry consistently

Most units put consistent emphasis on data entry through constant reminders by senior staff. In one unit, the senior nurse customizer walked the unit each morning reminding people of new data fields and asking for feedback on improving the CIS. In another unit, monthly sessions were held by the clinical customizer to discuss the data and demonstrate ways to improve entry. In a third unit, an online suggestion form was regularly used. These approaches, while felt to be highly successful by the clinical customizers, are time consuming and require substantial senior nursing leadership.

#### Structuring free text data

Free text data, although often preferred by health professionals, is not machine readable and needs to be structured to facilitate automatic extraction. Some units allowed the entry of both free text and structured documentation, but staff complained that it was difficult to know where to find the documentation of care details. One unit, in contrast, employed a number of strategies to utilize both types of data, but in clearly-defined contexts. The nursing flow sheet used pick lists to structure data, for example, which could then be annotated with free text and the medical record broke documentation up into specified free text areas to enable partial extraction of the free text data. This strategy enables the use of the data for both direct clinical care and process improvement, but requires some implementation thought on the part of the customizers.

#### Supporting manual extraction

It was often not possible to extract data automatically for clinical and service audit without adding more data entry fields. To avoid this, units had developed a number of techniques to facilitate manual extraction by those who wanted the data. These included: narrowing down the possible patients to be viewed to the most relevant 100 or so, particularly in very large databases; identifying potential patients in real time, for example, ones with ventilator-associated pneumonia (VAP), and alerting the duty consultant to check; and using hospital databases from other departments to cross-reference a data set. While these techniques could be useful, they required technical skills that were not available in all units as well as more time on the part of the person carrying out the audit.

#### Supplying visual representations of data

We saw the use of visual displays to support manual data extraction. Some units, for example, created specific views to support health professionals carrying out regular audits that required a large number of pages to be viewed in the CIS. The view pulled all the information needed into one screen for easy clinical judgment, such as a pharmacist retrospectively verifying the number of inappropriate drug prescriptions. Another unit used graphical representations of data to explore clinical problems, such as the relationship between filter clotting and other variables. These approaches were popular as they suited the existing skills sets of both the customizer and the health professional carrying out the audit, but worked best for regular or large projects.

#### Compensating with IT skill

Some units compensated for data input issues by manipulating data once it had been extracted from the CIS database. For example, they might use another software program to highlight and clean inappropriate values, discard missing ones, and eventually reformat the data so that calculations could be performed with it. The challenge of writing more complicated queries to extract large amounts of data was often outsourced. Each unit, for example, bought in outside expertise, either from the vendor or third party suppliers, to develop add-on modules to collect the data needed for government purposes, such as the Intensive Care National Audit and Research Centre and the Critical Care Minimum Data Set.

## Discussion

All units in this study faced difficulties in using CIS data collected during patient care for clinical and service audits that could contribute to clinical process improvement. Contrary to their own prior expectations, units in this study had been unable to derive immediate benefit from their CIS data after purchasing a CIS. Health professionals had to undertake extra work to repurpose the data either through entering it differently than would be necessary for direct clinical care or manipulating into a machine-readable format for audit purposes. Units were confronted with a tension over who was responsible for this extra work of producing audit data.

Units responded by developing strategies that motivated data entry by health professionals or mitigating its need through reformatting data in other ways. We identified 11 strategies that clinical customizers can use in their own units. Although the specific implementation varied from unit to unit depending on the CIS capabilities and the skill and time resource available, each strategy category was seen in at least three units. This suggests that they are neither CIS nor unit dependent. As such, these strategies can provide inspiration for units looking for ways to increase their usage of CIS data for clinical process improvement.

Many of the strategies relied on distributing the extra work between a number of roles so that the responsibility of data entry and reformatting did not rest entirely on front-line health professionals while carrying out their care duties. For example, clinical customizers rebuilt interfaces to make data collection easier, auditors worked from semi-extracted data to reduce data entry, and IT companies provided solutions for difficult tasks of data reformatting. The distribution of this extra work was not achieved in the same way in all units, but emerged in each over time as they negotiated their own circumstances, resources, and priorities. Distribution of extra work in such situations is an essential process that needs recognition.

It is notable that many of the strategies do not rely solely on sophisticated technology, but on human skill in its application to work processes. Our analysis indicates that clinical customizers play a pivotal, if often unrecognized, role in both shaping the CIS and its technological capabilities and in supplying leadership within the unit. They carry out much of the redistributed work. Given the need for a detailed understanding of the work processes and staff attitudes, it is perhaps not surprising that most people in this role were senior nurses with extra IT training. Providing appropriate recognition of this role and necessary training is likely to have a substantial influence on units' capabilities to develop appropriate strategies to use the CIS productively for both direct clinical care and clinical process improvement.

These findings provide some practical guidance on how units might repurpose the data that they collect during clinical care for clinical process improvement. Unlike previous studies, which articulate the problem of extra work and associated issues of system failure [5-9,19], this study suggests a more positive direction of enquiry, by considering strategies that foster compromise between different data needs. The ethnographic methods used to produce such findings however, can say little about the particular impact of a given strategy and is not able to ‘verify’ that it works beyond interviewees’ suggestions that it did so.

We would suggest that these findings are relevant to all health settings with the following characteristics: multidisciplinary teams; user-customizable CIS; and settings using their own data for clinical process improvement. For example, similar strategies might be used in hospital-based services, community services, or mental health services as advanced CIS start to become available in these less data-intensive environments [20]. These findings are not directly relevant to settings that give data to others to analyze, either for performance monitoring or to gain greater data manipulation capabilities. Such situations have the additional issue of data ownership, which has been explored elsewhere [21], as well as access to a set of skills and expertise not usually available in a clinical unit [22].

The results have several implications for those providing resources to health settings that are using their own data for clinical process improvement, both larger governing bodies, such as hospitals and NHS funding bodies, as well as policymakers. First, the process of distributing the extra work described above is not a standardized process and will take units time. In our study, units took about 4 years before they began to extract data regularly. Second, financial resources are needed to support those who have had work redistributed to them, such as those carrying out audits, doing customization work, or paying IT contractors. Units reported that little recognition was given to the efforts required in repurposing data, and compensation was never offered for the clinical customization time needed.

In addition to recognizing the costs of repurposing data, it is important to consider the role leaders might play in encouraging unit development that facilitates the use of CIS data for clinical process improvement and other secondary uses. In units in which research nurses were plentiful, but IT skills less so, nurses were used to input and extract data, as it was organizationally easier in the short term and more reliable to use nurses rather than invest in customization expertise. This would not seem to be desirable in the long term, however, if secondary use of data is to be promoted across the sector as a whole. Leaders can provide incentives to move towards greater automation of repurposing data.

## Conclusions

‘You can’t just push a button’ to gain data from the CIS for secondary purposes, as one unit lead mourned. As this study has illustrated, extra work is required to enter and reformat data, which must be distributed across multiple roles in a unit. In particular, clinical customizers play a substantial part in this process. Our findings indicate that adequate time, appropriate financial support, and motivation for acquiring the necessary skill can be built into policies to reap the value of data in CIS and enable the policy goals of using data for secondary purposes for clinical process improvement.

## Abbreviations

CIS: Clinical information system; ICU: Intensive care unit.

## Competing interests

All authors declare that they have no competing interests.

## Authors’ contributions

CM, MJ, and AV contributed to the design of the study. CM carried out the contextual interviews. CM and RJ coded the data. CM wrote the first draft of the manuscript. All authors were involved in revising the manuscript and have given final approval of the version to be published. AV is the guarantor.

## Authors’ information

CM is a post-doctoral researcher in eHealth. She holds degrees in both Anthropology and Computer Science. MJ is a lecturer in Organisational Systems and Healthcare at the University of Cambridge. RJ is a Human-Computer Interaction expert with a PhD in Computer Science. AV is a consultant in Anaesthesia and Intensive Care at Papworth Hospital NHS Foundation Trust.

## Pre-publication history

The pre-publication history for this paper can be accessed here:

http://www.biomedcentral.com/1741-7015/11/103/prepub

## Supplementary Material

Additional file 1 Data supplement. Click here for file
