# Supplementary material for: ‘You can’t just hit a button’: an ethnographic study of strategies to repurpose data from advanced clinical information systems for clinical process improvement
Source: BMC Med. 2013 Apr 10;11:103. doi: 10.1186/1741-7015-11-103 (PMC3635898; doi:10.1186/1741-7015-11-103)
Supplement: Additional file 1 — Data supplement. [file 1741-7015-11-103-S1.pdf]

DATA SUPPLEMENT

Table 3

Row 1 – Data entry not needed for direct clinical care: Data entry needs augmenting

Example:
Doctors are asked to enter diagnosis at the time of a patient's admission. Diagnosis is not always known at this time and of little importance to the doctors who base their work on active issues. For this reason, it often does not get filled in correctly or at all. Its completion is an augmentation of data needed for direct clinical care.

Data context:
Observation taken from researcher's field notes describing a user forum sponsored by one of the CIS vendors.

Data:
Two consultants who direct the ICUs in which they work commiserated that they cannot get any decent data out of their CIS. I asked why they had so much trouble. With an outpouring of frustration, both said that their colleagues did not always enter diagnosis so that it was hard to filter the patients.I challenged them as to whether they themselves always entered the diagnosis. They both insisted that they did unless it was not known.

Data context:
Quotation from a consultant in Unit D discussing how he navigates the CIS. His second statement refers to the problem list.

Data:
Respondent: Well there is a problem list that tells you all the ongoing issues, if you see what I mean, but very much a superficial thing that's primary function is designed for coding when it comes to discharge as opposed to updating the clinician. So no, there isn't really any, if you like summary of active issues and what's happening with those enumerated in any reliable way.

I mean this is what people need to put in when somebody gets admitted, what the primary diagnosis is and obviously their background problems. Any new problem [inaudible 00:27:52], now this is just all over the place.

Row 2 – Data entry not needed for direct clinical care: Duplicate data entry needed

Example:
Junior doctors are asked to enter past medical history twice: into the admission form and into an audit form. They find this frustrating and time-consuming and assign it a low priority with the ramification that it does not always get done.

Data context:
Quotation from a junior doctor in Unit A following several queries from the researcher as to why so many of the boxes on the form were blank. In the first statement he is explaining the audit form which is submitted to ICNARC: Intensive Care National Audit and Research Centre. In the third statement he is discussing how data in the audit form does not automatically populate the clinical notes requiring him to duplicate data entry.

Data:
Respondent: Because it requires you to input lots of data which to us clinically is not very relevant. A lot of it is for the nurses. I mean we do it and you see how it is but it doesn't really help. For example we put all the past medical history that we want to know about the patients history in here, we just write it and that comes through. This is something everyone can read. This bit in red, enter past medical history, ICNARC data, so ICNARC is this auditing thing, so I've never seen it and it takes ages to input it on that particular thing but you can't see it on the clinical thing, so you'd have to write it anyway but it won't let you not do this, if that makes sense.

So that's very annoying and it's not particularly helpful. You end up just yes, no-ing questions and if you want to put like, do they have a heart problem? You know they have loads of heart problems and it's quite difficult to annotate it, it's quite time consuming, so that's not good.

...

No, this is just one example of past medical history and things like medications, so medication, another example, you can put it all here but it doesn't come up onto the general notes so you have to cut and paste it into that box. It's daft, where it could just easily...and that's basically what I'm trying to say about this bit. If you could see what they want you to put in there on the screen then you wouldn't have to write it up here and you wouldn't feel like you're doing it for something that you think is pointless.

Row 3 – Data entry not needed for direct clinical care: Quality control of data needed

Example:

Nurses are asked to validate measures captured automatically from the monitors. This ensures that values are accurate and not distorted (e.g. because the patient moved). Their accuracy is of little value to nurses who can clearly see they are wrong and the correct values adjacent. They do not always correct mistakes, especially if busy. This causes considerable problems at audit time when highest and lowest parameters are searched for.

Data Context:

Quotation from the nurse clinical customizer in Unit D discussing why he looks over the data that is automatically extracted from the system. The term obs refers to observations, the taking of vital measures at regular intervals.

Data:

Respondent: The one thing about an electronic system I think is that people can become complacent in the sense that when obs come across every hour, this is an example. They have to be validated, not just accepted and nurses will validate obs, blood pressures is at 322 over 60, [inaudible 00:37:14] woman moved her arm on the hour or the gentleman moved his arm.

So yeah, you do have to still be a nurse to see...like everything nowadays, you need to look to make sure it's roughly right. You can't just...I mean people do do that and that's probably just a time thing, you know, rushing around

I don't always take their word for it, I always check it because it's mistakes and people put the wrong information in or you've got the admission date when they came in, when they were admitted and you've got the first set of highest and lowest obs for the first 24 hours, which this woman hasn't got, bad example. Let's go to that person. So the lowest systolic, [inaudible 00:13:23], the highest systolic and that's the first 24 hours that they came in, which is what ICNARC wants.

Data Context:

Observation from a discussion with a charge nurse in Unit D when walking around the unit for the first time as captured in the researcher’s fieldnotes.

Data:

The charge nurse took me on a tour of the unit and pointed out what people were doing. We stopped at one bedside while the charge nurse discussed a clinical issue with the bed nurse. I looked at the screen of the clinical information system orienting myself to how this particular system organized data. I noticed that two of the data rows had extreme values in them. I queried why this was after leaving the bedside. She said that some nurses did not check, or perhaps did not care, what they validated. In their defense, she pointed out that some did not see the point in correcting values when they could easily see the normal values on either side of the extreme one. She then admonished the nurse’s lack of attention saying that it causes problems for ICNARC data collection as these extreme values were often picked up as the highest values. But, she finished, the bedside nurses, do not really understand this.

Row 4 – Data not machine- readable: Data not specifically recorded

Example:
An audit for septic patients is not possible because it is not a diagnosis. The computer can only identify those with bacterial pneumonia as a main diagnosis. In contrast, a nurse can easily tell if a patient is septic from looking at the clinical record.

Data Context:
Quotation from a charge nurse in Unit D whose role includes doing one or two audits per week. She explains why she does audits manually rather than having the data extracted automatically for her.

Data:
Respondent : ...but the problem with that is apparently, he asks for specific information and only if that specific information is somewhere on the system can it pull it out.

So for example I do a lot of work on septic patients. Now it would be very easy if [the nurse clinical customiser] could ask the computer to pull out all the patients with sepsis, but unfortunately it's not as easy as that because on the diagnosis which is on the doctor’s page, the doctors put in a diagnosis, an ICNARC diagnosis, they'll put in bacterial pneumonia. The patient may be septic as well, not necessarily but maybe, but they wouldn't actually put sepsis as a diagnosis on there. So the computer would pull out all the patients who have a diagnosis of sepsis, but not all the patients with sepsis would have that written up there, so that's the problem. I quite like trawling through [the patient records].

Row 5 – Data not machine- readable: Data not in appropriate format

Example:
Clinicians prefer to write their notes in free text to more aptly express the issues of focus to their colleagues. This can make diagnosis, problems, and actions difficult to extract from CIS data.

Data context:
Quotation from the medical clinical customiser in Unit B about preferences for the medical notes form. This data reflects sentiments found across health professionals that most preferred free text for their notes, particularly doctors.

Data:
Respondent: Spent time to design the form, a lot of discussions but complex forms came along and they spend weeks and weeks and suddenly one of my colleagues say, "But why don't we just do a very simple page of paper and just the headings should be the same for all and the rest people can write whatever they want." People say, "Oh yes, that's really what we want. So we don't want to be down to tick boxes and drop down lists etc, we just want something that goes around with what we do clinically and makes sense when we read it back, etc,"

Data context:
Quotation from the audit nurse in Unit C during a discussion of the two types of data fields he queries: structured (e.g. drop down menus) and unstructured (e.g. free text). He refers first to the flow sheet which looks like a spread sheet with value names on the left and time across the top. Health professionals can enter numeric values or choose from a drop-down menu. The second form he discusses (after ...) is the medical documentation which is a screen broken down into boxes for writing of free text (e.g. medical ward round)

Data:
Respondent: Yeah, so the flow sheet, the front end, is very controlled data. It's usually a case of you can put a number in or pick from a pick list. From an audit perspective that is wonderful because we know exactly what we're looking for, but that's the flow sheet. So outside of that then we will have notes...

From an audit perspective it's not too bad because we've got specific things that we can look in but then what we obviously run into the problem of is spelling mistakes and everything else, abbreviations etc. So we've moved from the field where, for example,

position of the patient, you have to choose from 12 choices. Here you could type whatever you wanted so it becomes a little bit more difficult to audit against.

Data context:
Quotation from the nurse clinical customiser in Unit C during a discussion of the design of forms. She discusses different types of communication that needs to be captured.

Data:
Respondent: Flow sheet is very easy auditable and I'm sure [the audit nurse] will have talked extensively about that. Forms are much harder to audit particularly when you get into free text but obviously they have much more of a qualitative feel rather than a quantitative feel about them.

Interviewer: How do you balance the choices about using forms versus using flow sheets in terms of the clinical work and the data needs?

Respondent: You've got to think about what you're trying to capture. So much in ICU is quantitative, quantitative data but there are lots of things that need that qualitative feel so I would expect the majority of the documentation problems to be quantitative but there are things like, you know, how a patient's feeling, how they look, etc, etc. All those things. Physicians opinions, nursing opinions, multi-disciplinary team advice, whatever – all those things are really difficult to quantify. Pick lists don't work, you know, when a patient's newly admitted. Some units have managed to do it with a sort of a fully structured pick list. I don't think that works personally. It think it's quicker and more accurate for the medical staff to be able to document how they would normally.

Row 6 – Data not machine- readable: Terminology not consistent

Example:
Doctors use a range of terminology for common problems. For example, out of hospital arrest can be termed: cardiac arrest, cardiac standstill, cardiac asystole, and ventricular fibrillation. This makes it very difficult for the computer to search for data related to out of hospital arrest as it cannot assimilate all of the related terms easily as a doctor could do.

Data context:
Quotation from medic clinical customiser in Unit A discussing a design issue which makes it difficult to control vocabulary in the problem list with the ramification that querying by diagnosis becomes problematic.

Data:
Respondent: I mean if you just look at this, if you just want to query cardiac arrest you have to look at all different possibilities because trainees can enter free text here, you can just add new and change it.
Interviewer: Okay, so it's hard to keep consistent.

Respondent: Yeah, that's because people have been allowed to put things in. If somebody puts cardiac arrest and it doesn't accept it [inaudible 00:28:26] heart stopped or something like that and then when you actually query it I've got six different variables for just out of hospital arrest. That's [inaudible 00:28:39] cardiac arrest, cardiac standstill, cardiac asystolly, venticular fibrilliation, [inaudible 00:28:45] and things like that.
